# Developing the surgeon-machine interface: using a novel instance-segmentation framework for intraoperative landmark labelling

**DOI:** 10.3389/fsurg.2023.1259756

**Published:** 2023-10-23

**Authors:** Jay J. Park, Nehal Doiphode, Xiao Zhang, Lishuo Pan, Rachel Blue, Jianbo Shi, Vivek P. Buch

**Affiliations:** ^1^ Department of Neurosurgery, The Surgical Innovation and Machine Interfacing (SIMI) Lab, Stanford University School of Medicine, Stanford, CA, United States; ^2^ Centre for Global Health, Usher Institute, Edinburgh Medical School, The University of Edinburgh, Edinburgh, United Kingdom; ^3^ Department of Computer and Information Science, School of Engineering and Applied Science, University of Pennsylvania, Philadelphia, PA, United States; ^4^ Department of Computer Science, University of Chicago, Chicago, IL, United States; ^5^ Department of Computer Science, Brown University, Providence, RI, United States; ^6^ Department of Neurosurgery, Perelman School of Medicine at The University of Pennsylvania, Philadelphia, PA, United States

**Keywords:** artificial intelligence, intraoperative guidance, machine learning, surgical guidance, spine, arteriovenous fistula, surgeon-machine interface, global neurosurgery

## Abstract

**Introduction:**

The utilisation of artificial intelligence (AI) augments intraoperative safety, surgical training, and patient outcomes. We introduce the term Surgeon-Machine Interface (SMI) to describe this innovative intersection between surgeons and machine inference. A custom deep computer vision (CV) architecture within a sparse labelling paradigm was developed, specifically tailored to conceptualise the SMI. This platform demonstrates the ability to perform instance segmentation on anatomical landmarks and tools from a single open spinal dural arteriovenous fistula (dAVF) surgery video dataset.

**Methods:**

Our custom deep convolutional neural network was based on SOLOv2 architecture for precise, instance-level segmentation of surgical video data. Test video consisted of 8520 frames, with sparse labelling of only 133 frames annotated for training. Accuracy and inference time, assessed using F1-score and mean Average Precision (mAP), were compared against current state-of-the-art architectures on a separate test set of 85 additionally annotated frames.

**Results:**

Our SMI demonstrated superior accuracy and computing speed compared to these frameworks. The F1-score and mAP achieved by our platform were 17% and 15.2% respectively, surpassing MaskRCNN (15.2%, 13.9%), YOLOv3 (5.4%, 11.9%), and SOLOv2 (3.1%, 10.4%). Considering detections that exceeded the Intersection over Union threshold of 50%, our platform achieved an impressive F1-score of 44.2% and mAP of 46.3%, outperforming MaskRCNN (41.3%, 43.5%), YOLOv3 (15%, 34.1%), and SOLOv2 (9%, 32.3%). Our platform demonstrated the fastest inference time (88ms), compared to MaskRCNN (90ms), SOLOV2 (100ms), and YOLOv3 (106ms). Finally, the minimal amount of training set demonstrated a good generalisation performance –our architecture successfully identified objects in a frame that were not included in the training or validation frames, indicating its ability to handle out-of-domain scenarios.

**Discussion:**

We present our development of an innovative intraoperative SMI to demonstrate the future promise of advanced CV in the surgical domain. Through successful implementation in a microscopic dAVF surgery, our framework demonstrates superior performance over current state-of-the-art segmentation architectures in intraoperative landmark guidance with high sample efficiency, representing the most advanced AI-enabled surgical inference platform to date. Our future goals include transfer learning paradigms for scaling to additional surgery types, addressing clinical and technical limitations for performing real-time decoding, and ultimate enablement of a real-time neurosurgical guidance platform.

## 1. Background

Intraoperative application of Artificial Intelligence (AI) is a rapidly advancing area in surgical innovation. AI technology offers various capabilities within the operating room, such as automating workflows and aiding in intraoperative decision-making (1, 2). The ultimate objective is to leverage AI’s potential to learn, interpret, predict, and solve problems by training Machine Learning (ML) algorithms. These algorithms can process vast amounts of real-world data and guide decisions comparable to those of expert surgeons (3). We have introduced the term Surgeon-Machine Interface (SMI) to describe the advanced and innovative fusion of surgeons and machine interfaces, creating a new realm of collaboration. Computer Vision (CV) plays a pivotal role in facilitating interaction with intraoperative data, enabling machines to comprehend surgical images and videos (4). It also serves as the foundation of current endeavours in intraoperative landmark guidance. However, the availability of literature and regulatory-approved devices for real-time AI-based anatomical landmark labelling is limited, indicating that this technology is still in its early stages (5, 6). Nevertheless, recent advancements in artificial neural networks (ANNs), a subfield of ML and the backbone of deep learning (DL), show promise in enabling AI to achieve even higher levels of performance in this field (5, 6).

In a surgical setting, there are two crucial CV tasks: recognition and tracking. Object recognition employs machine learning (ML) to identify objects within an image, similar to human perception. When combined with an object localization algorithm, object detection can be achieved. This algorithm generates a bounding box that encompasses the object and provides a label for it. However, in surgical applications, where anatomical structures have intricate contours and unclear boundaries, a single bounding box may not accurately capture the desired area (7). Object segmentation addresses this limitation by producing pixel-wise masks that offer a detailed labelling of individual objects within an image (8). There are two types of segmentation: semantic segmentation groups similar pixels into a single classification, while instance segmentation distinguishes and segments each individual entity. In essence, instance segmentation allows for the pixelwise classification of individual objects within a surgical field, whether they are anatomical structures or surgical instruments. Although, it is the most preferred recognition technique for intraoperative guidance (9), previous attempts have been limited to segmenting rigid surgical instruments (10), as opposed to anatomical structures, which are often characterised by semi-rigid boundaries and thus pose a more difficult segmentation task.

Although few, there have been some platforms and clinical evidence in general surgery that attests to the accuracy and the significance of AI software in an intraoperative setting (7, 9, 11–14). Neurosurgery, at the forefront of cutting-edge technology, has witnessed numerous advancements in AI applications; however, these applications are limited to surgical phase recognition (15), detection and surveillance (16), diagnosis (17, 18), endovascular navigation (16), training and preoperative planning (2, 16, 19–21), intraoperative imaging, and workflow automation (22). To our knowledge, there is no other literature or technological reports that demonstrate a scalable surgical video analysis system in neurosurgery. In this study, we aim to demonstrate the most advanced surgical CV architecture to-date, and for the first time applied to a neurosurgical context. Although a fully functioning SMI will incorporate real-time implementation with a user interface, in this manuscript we seek to introduce the core technology for the conceptualisation of our future real-time enabled SMI. We demonstrate our custom instance segmentation core architecture and prediction model in a proof-of-concept for open spinal dural arteriovenous fistula (dAVF) surgery.

## 2. Methodology

### 2.1. Developing the AI framework

**Figure 1:**
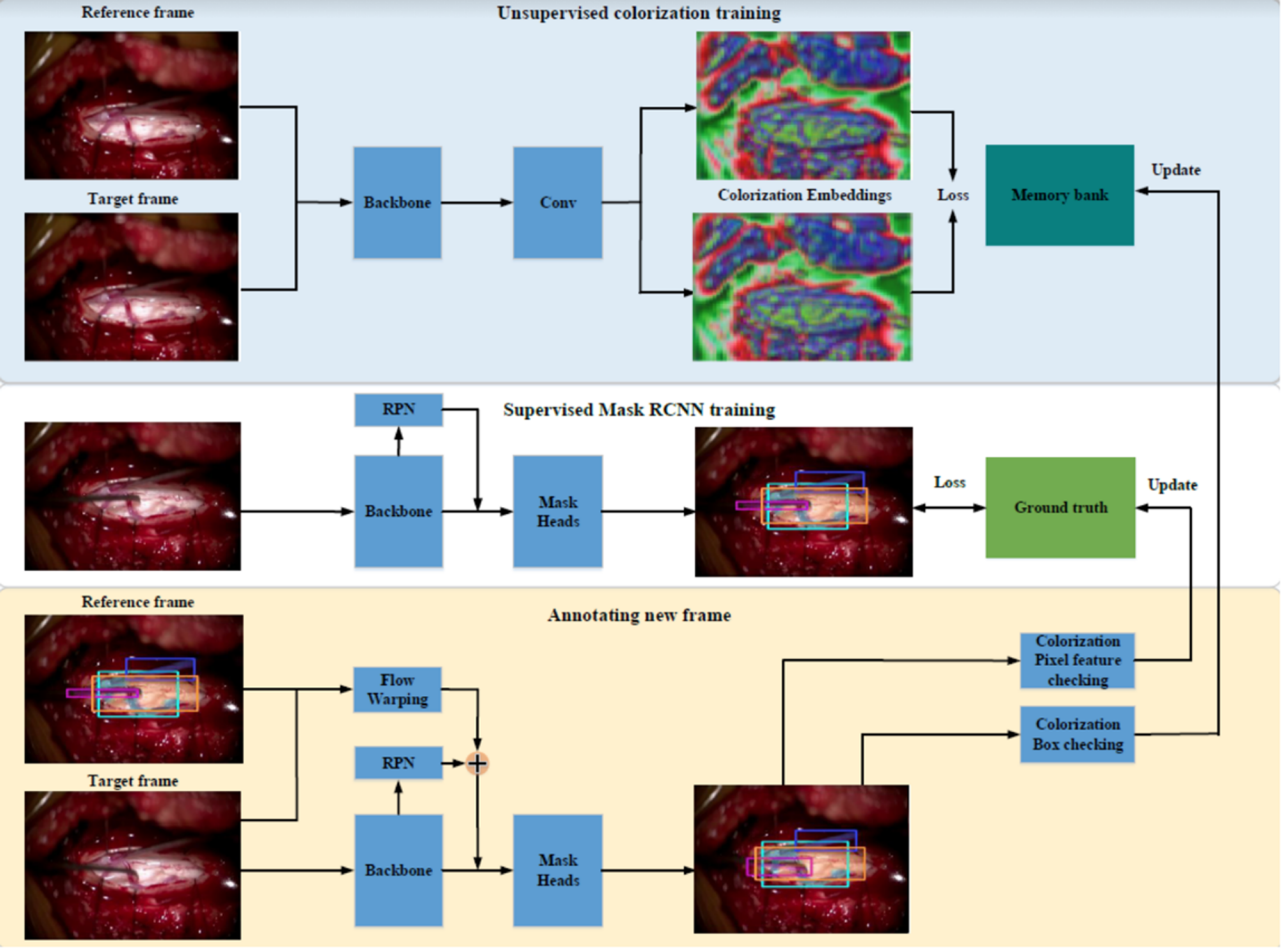
The initial mask-RCNN architecture.

**Figure 2:**
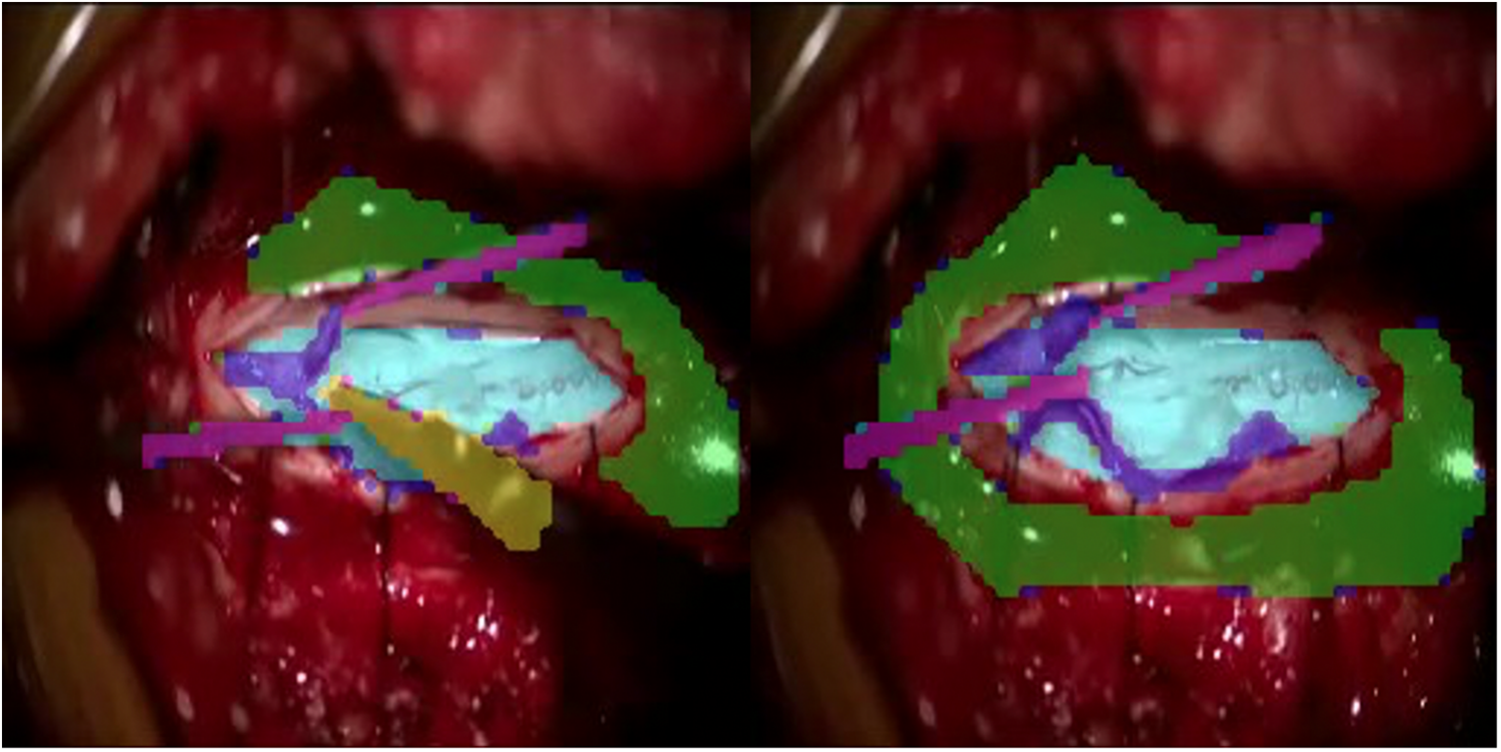
Poorly demarcated annotation using colourisation technique.

Our first prototype framework consisted of three parallel components (Figure 1). (1) A single frame would be processed through Mask Region-based Convolutional Neural Network (MaskRCNN) training, producing class-agnostic boxes. We train MaskRCNN in a supervised manner on a few annotated frames. (2) We augment the prediction capability of MaskRCNN by including temporal information from Colourisation, an unsupervised technique to learn the frame-wise feature correlation. Specifically, we extract the unsupervised feature flow from Colourisation. Then we use it to propagate the instance mask from the previous frame to the consecutive frames serving as complementary predictions. (3) Propagation *via* feature flow also yields pseudo ground truth instance segmentation to further improve our Mask R-CNN model. To filter out the noisy label, we build a rolling updated memory bank to collect high-quality predictions and score the incoming pseudo prediction. Only examples above the threshold will be used to fine-tune the Mask R-CNN model. If few annotated labels are present, only process (1) and (2) will take place. This architecture rendered inadequate results with anatomical landmarks being poorly demarcated as shown in Figure 2. The major issue was that the embedding flow from colourisation is biassed to the low-level texture. Tracking without mid and high-level features is fragile in the challenging case.

**Figure 3:**
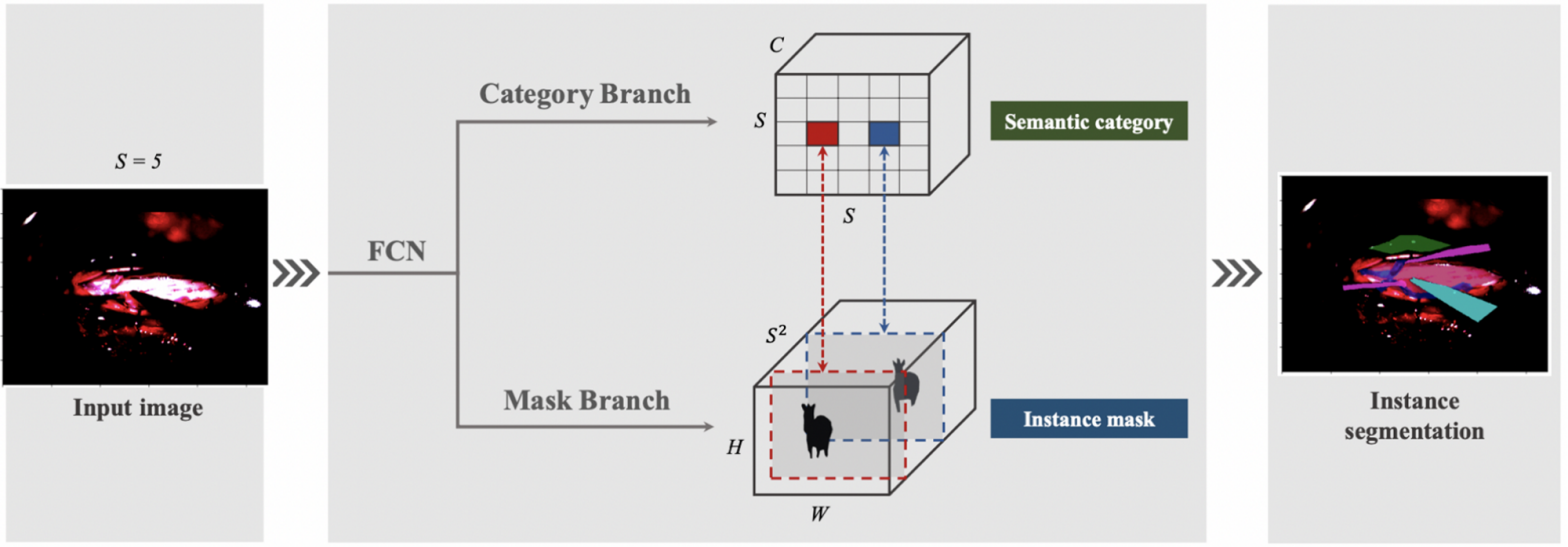
Initial SOLOv1 architecture.

Our next attempt was to utilise SOLOv1 architecture with Kanade-Lucas-Tomasi (KLT) tracking system, as Figure 3. In SOLOv1, the image is divided into a grid and objects are located for every cell in the grid. Instance category and instance mask are computed in parallel across the grid. Since frame-by-frame instance segmentation has a disadvantage of lacking temporal information we embedded a KLT tracking system into the segmentation system to recognize the objects moving across video. The tracking system generates initial feature points over the masks in the first frame. KLT feature tracker tracks these feature points crossing the video. The identity of a segmentation is determined by a majority vote over all the feature points.

**Figure 4:**
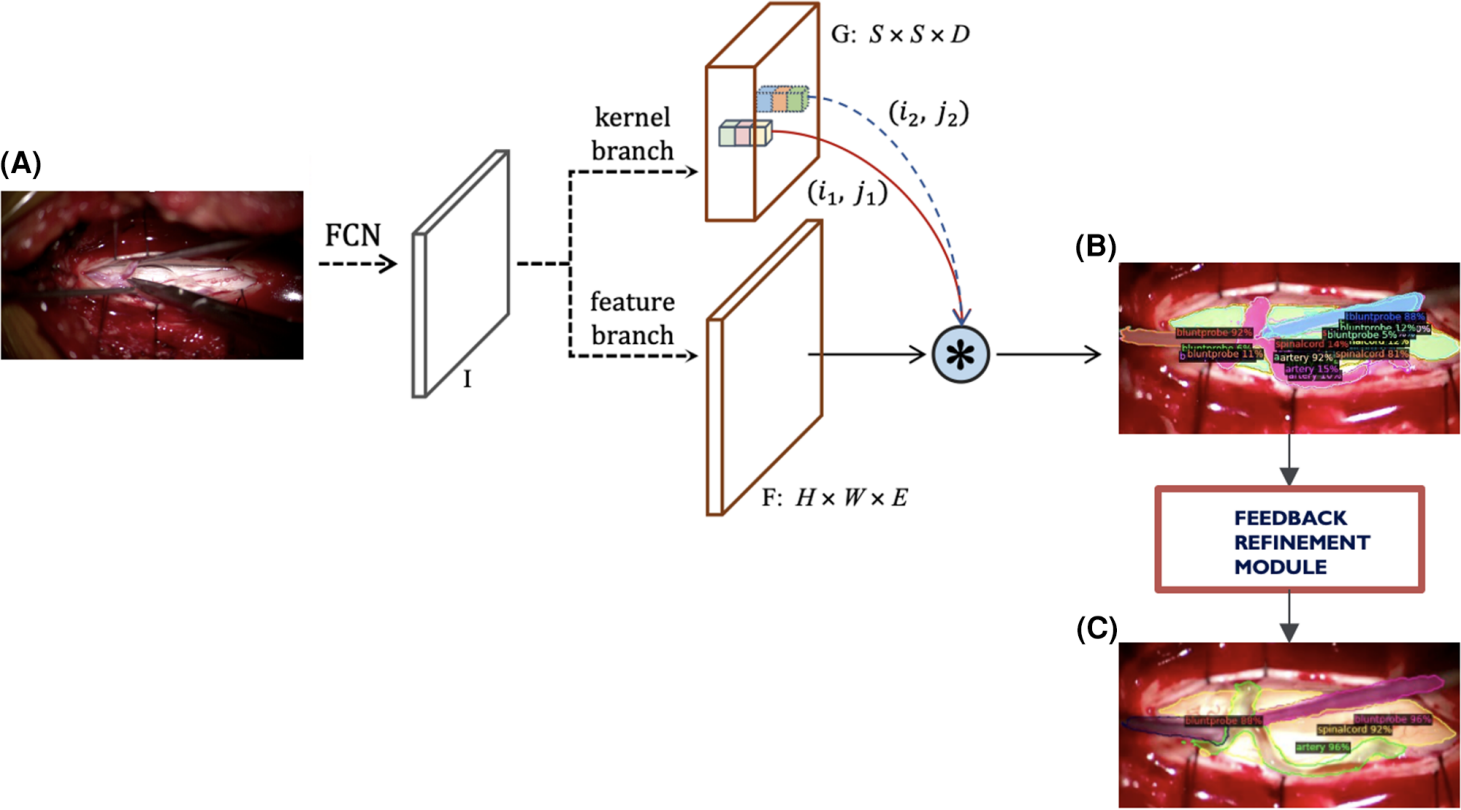
Current modified SOLOv2 architecture. (**A**) Input video frame, (**B**) Model output predictions, (**C**) Refined predictions.

To account for the challenges faced with our previous instance segmentation frameworks, we developed a novel segmentation algorithm (Figure 4) based on the state-of-the-art SOLOv2 architecture, a dynamic and fast framework for real-time object detection. In SOLOv2, the mask head is further decomposed into 2 branches, namely, feature branch and dynamic convolution kernel branch. Instead of forwarding the feature map directly through another layer of convolution, the feature map is used to learn a dynamic convolution head, which is a kernel map that convolves with the feature map to output the final mask prediction. However, in our experiments we face a practical limitation of the SOLOv2 framework and obtain many false positive detections. To alleviate this issue, we customise the framework to refine this low confidence but plausible detections into high confidence and obtain higher performance in instance segmentation (Figure 4).

All algorithms were constructed on Ubuntu 20.04.4 LTS (x86_64; Canonical Ltd., London, United Kingdom) and developed using the Detectron2, a Pytorch object detection library in Python (Language).

### 2.2. Dataset preparation

A single test video of spinal dAVF surgery was recorded with Zeiss Opmi Pentero 800 (Carl Zeiss AG, Jena, Germany) at The Hospital of the University of Pennsylvania. This video constitutes 8,520 frames. A trainee neurosurgeon (RB) annotated 133 frames using Computer Vision Annotation Tool (CVAT 7.4.0; Irvine, California, United States). 133 frames were sampled every 30 frames within the first 1.5 min of the surgical video (in-domain). These frames consist of the operator dissecting the arachnoid and separating the two abnormal dorsal spinal arteries (part of the dural AVF) with a blunt probe, dissector, and micro-scissors followed by the temporary clipping a dorsal spinal artery with an aneurysm clip after. Any ambiguities with anatomical structure were clarified by an attending neurosurgeon (VPB).

### 2.3. Statistical analysis

To assess segmentation accuracy and computing speed, we applied different frameworks to the single surgical video and validated it across 85 frames. These frames were uniformly sampled every 3.33 s from entire 4.73 min of the video. The validation set comprised of 27 frames which were in-domain and 58 of these frames that were out-of-domain frames, which was a hold-out test set as a temporal partition from the same video. The in-domain frames constitute unlabelled frames within the period of the training set as opposed to the out-of-domain frames that were unlabelled frames after the last time period of the training set. In these out-of-domain frames, the surgeon irrigated the surgical field, suctioned pools of blood outside the dura, manipulated the arteries with a suction and blunt probe, and finally removed the aneurysm clip with the applier. Instance segmentation was performed by MaskRCNN + Feature Pyramid Networks (FPN), SOLOv2 + R101DCN, SparseRCNN, YOLOv3, and our SOLOv2-based modified architecture. We then calculated certain metrics to measure precision for each of these architectures: mean Average Precision (mAP), and F1 score. Accuracy was tested on in-domain frames, which were part of the validation set, as well as out-of-domain frames which were not part of the test or validation set. Furthermore, we evaluated the inference time for the computing speed of each of the networks.

All these frameworks were processed in a single computer on Ubuntu 20.04.4 LTS (x86_64; Canonical Ltd., London, United Kingdom) with GeForce RTX 2080 Ti (NVIDIA, Santa Clara, United States) mounted. Statistical analysis was all performed using the Detectron2 (software).

## 3. Results

Our SMI framework outperformed any other known frameworks reported in literature for intraoperative landmark guidance in terms of accuracy and computing speed as shown in Table 1. F1-score and mAP of our model was 17% and 15.2% respectively, in comparison to the original SOLOv2 architecture which was 3.1% and 10.4%, YOLOv3 with 5.4% and 11.9%, and MaskRCNN with 15.2% and 13.9%, respectively. Taking into consideration detections that surpassed the Intersection over Union (IoU) threshold of 50%, our SMI had F1-score of 44.2% and mAP of 46.3%. This was followed by the MaskRCNN architecture with F1-score of 41.3% and mAP of 43.5%, YOLOv3 with 15% and 34.1%, and finally SOLOv2 with 9% and 32.3% respectively.

**Table 1:** Accuracy and computing speed of our SMI architecture and other commonly reported frameworks in literature.

	F1	F1 (iou 50)	mAP	mAP50	Inference time (ms)
SOLOv2	0.031	0.09	10.4	32.3	100
YOLOv3	0.054	0.15	11.9	34.1	106
MaskRCNN	0.152	0.413	13.9	43.5	90
Our SMI	**0**.**17**	**0**.**442**	**15**.**2**	**46**.**3**	**88**

Bold values are results from novel architecture.

**Figure 5:**
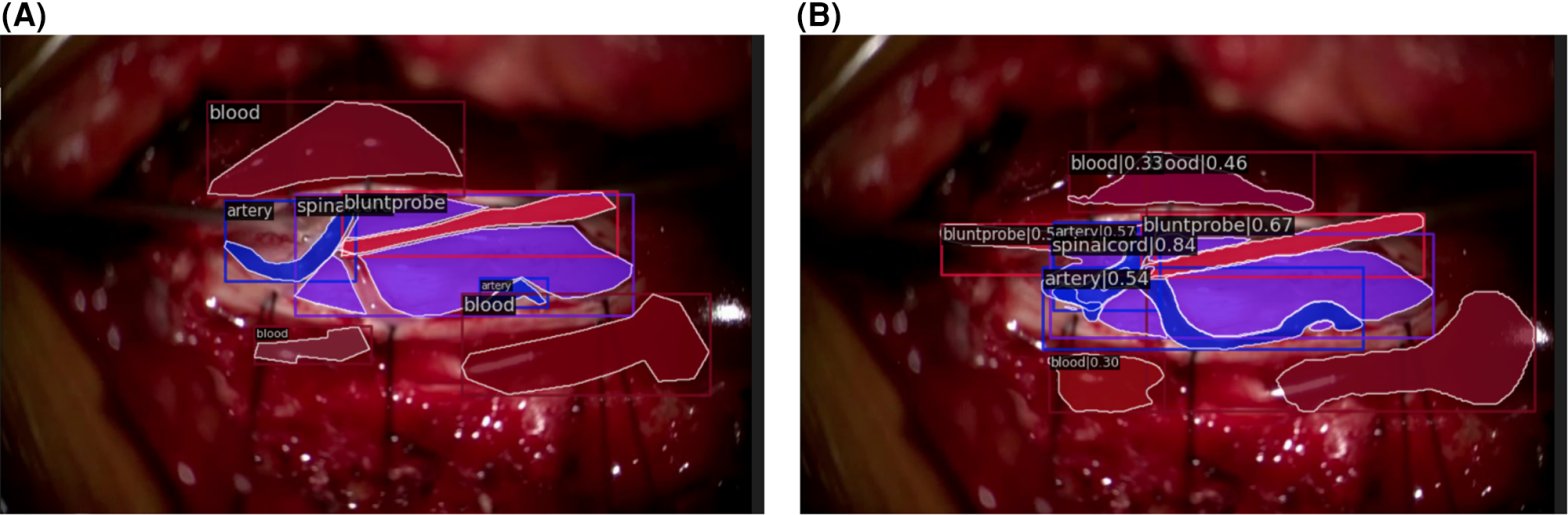
In-domain segmentation frames. (**A**) Ground truth frame of in-domain segmentation. (**B**) Prediction frame of in-domain segmentation.

**Figure 6:**
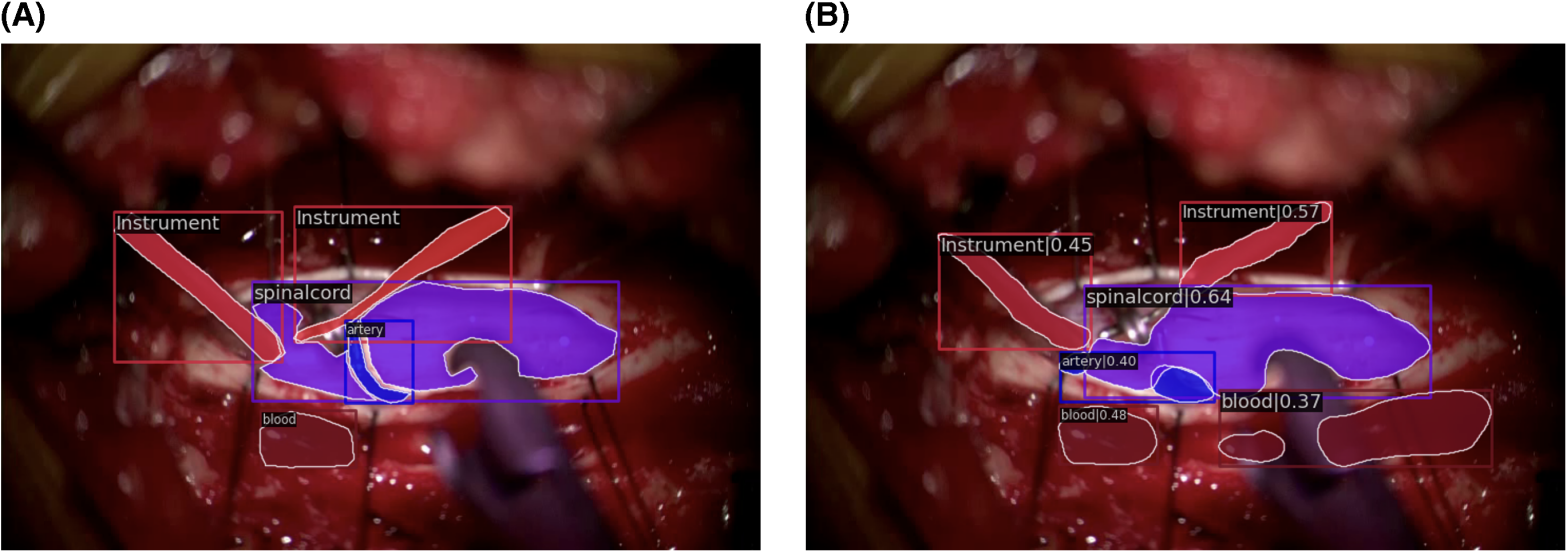
Out-of-domain segmentation frames. (**A**) Ground truth frame of out-of-domain segmentation. (**B**) Prediction frame of out-of-domain segmentation.

Qualitatively, our SMI architecture was successful in identifying objects in both in-domain frames (Figures 5A,B) and out-of-domain frames (Figures 6A,B), which indicates a good generalisation based on training on a quarter of the full surgical video. Our in-domain frame predictions demonstrated a high mAP score of >0.50 for anatomical structures and surgical tools and achieved >0.30 for distractions such as pool of blood. Most importantly, it was able to identify the blunt probe which was not annotated in the ground truth frame and was also able to predict a segment of the artery that was not annotated (Figure 5B). Identification of objects in out-of-domain frames also demonstrated visually promising results and high accuracy (Figure 6B). To highlight, the model was able to identify the suction as a different instrument in the out-of-domain frames, even though it has never seen the object before within the training set (Figure 6B).

The computation speed of our SMI was the fastest amongst all other frameworks (Table 1) with inference time of 88 ms, followed by MaskRCNN (90 ms), SOLOv2 (100 ms), and YOLOv3 (106 ms).

## 4. Discussion

This study introduces an advanced surgical platform that utilises instance segmentation for intraoperative guidance. The obtained results demonstrate the feasibility of our SMI framework for real-time application in spinal dAVF surgeries and its potential for adaptation to other neurosurgical cases. Our developed framework for the guidance system surpasses previously described frameworks in the literature in terms of precision and computational speed. Prior efforts in the field of general surgery demonstrated various elements of utility; however, none reports segmentation of both anatomical landmarks and surgical tools intraoperatively. Moreover, all segmentation efforts in general surgery are limited to YOLOv3-based bounding boxes and semantic segmentation techniques. Nakanuma et al. recently published a feasibility trial (J-SUMMIT-C-01) for a YOLOv3-based object detection framework to be used for intraoperative guidance in laparoscopic cholecystectomy (LC). Although they used the YOLOv3 framework, they were able to demonstrate an objective usefulness of an AI-powered surgical guidance platform (11, 12). In addition, Liu et al. provided supporting evidence that their YOLOv3 based framework identified anatomical structures within LC more accurately than their trainees and senior surgeons (13). Laplante et al., Madani et al., and Mascagni et al., on the other hand, utilised a semantic segmentation method to successfully determine safe and danger zones for surgical dissection and labelling anatomical structures relevant in LC (7, 14). Moving forward, Kitaguchi and his team developed an instance segmentation model but they have only classified surgical instruments in laparoscopic colorectal surgeries (9). In neurosurgery, Bouget et al. reports an attempt to identify intraoperative tools by an outdated method of semantic labelling and shape-based detection using supervised-vector machine (SVM) training (22), which has been further improved by Kalavakonda et al., with binary and instance segmentation approach (23). Therefore, our study reports the first and the most advanced use of instance segmentation in the surgical field to date.

Our extensive experience and previous failed attempts have significantly contributed to the development of a scalable segmentation framework in surgery. We have encountered challenges related to colourisation, the MaskRCNN architecture, SOLOv1, and KLT feature trackers, which have informed our understanding of these issues. After careful evaluation, we opted to modify the SOLOv2 architecture due to its superior performance compared to counterparts like YOLOv3 (24). Moreover, another compelling reason to use SOLOv2 was due to its ease of debugging. With SOLOv2, it becomes possible to visualize the features for each grid point, considering various kernel choices. This ability enhances the network’s expressive power, allowing for a deeper understanding of the underlying processes and facilitating effective troubleshooting. Ultimately, through extensive experimentation and framework modifications, we have successfully established a platform that facilitates the swift adoption of new segmentation frameworks for improved outcomes.

Additionally, we have gained valuable insights into the use of more objective metrics for instance segmentation. While mean Average Precision (mAP) has been commonly endorsed in clinical literature, its susceptibility to false positives makes it less suitable as a metric for instance segmentation in the surgical field (25). Instead, metrics such as the F1-score, precision, and recall provide a more accurate evaluation (25). Nakanuma et al. used the DICE coefficient as a metric for accuracy; however, we have incorporated this into our loss function (12).

**Figure 7:**
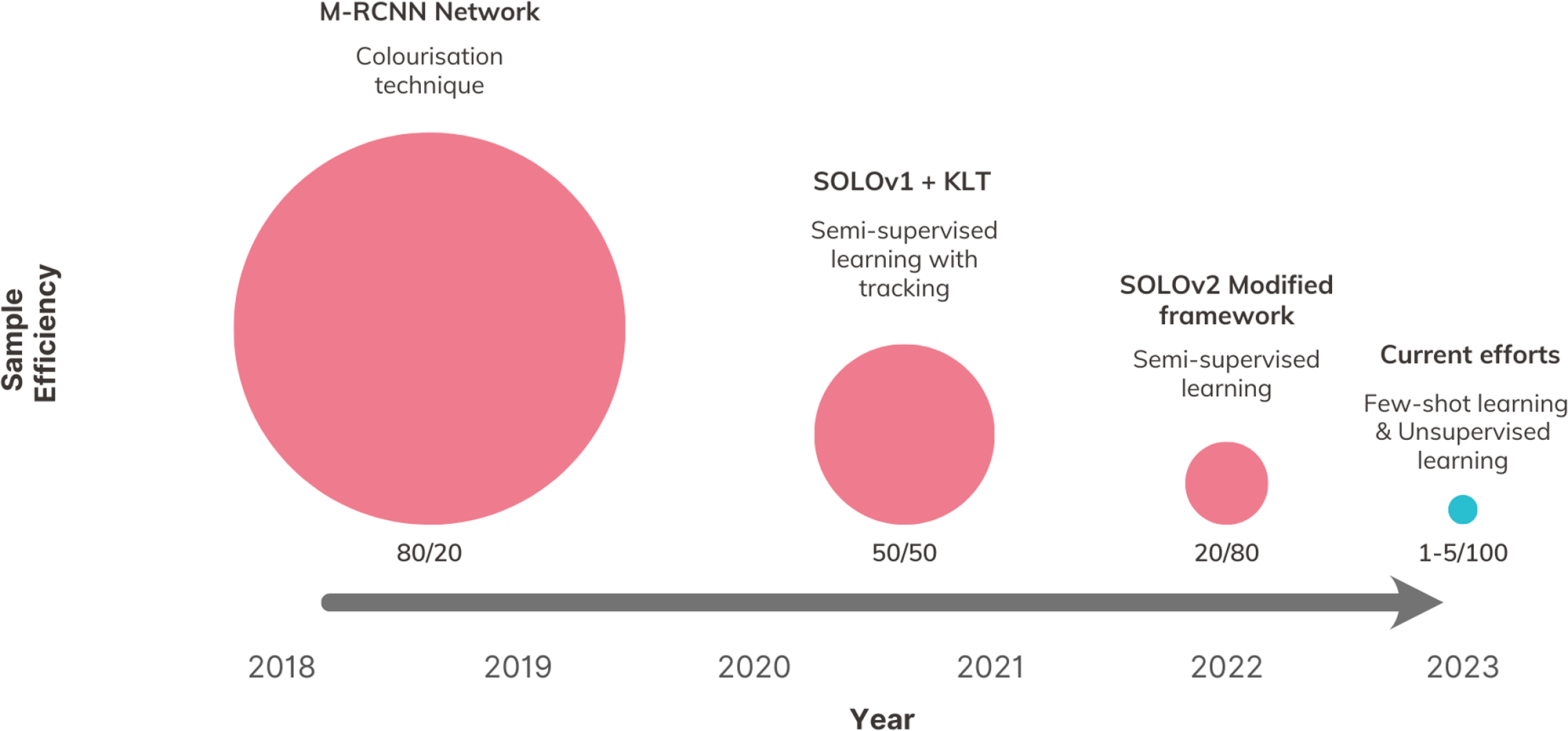
Comparison of sample efficiency across the versions of our SMI.

In this study, we present a novel comparison of sample efficiency among different intraoperative CV architectures, which, to our knowledge, is the first attempt of its kind in the literature. To measure sample efficiency, we calculated a ratio for each version of our SMI framework. This ratio represents the percentage of total available data used for training compared to the percentage used for testing, while achieving the same level of segmentation accuracy. Our findings reveal a positive trend in reducing the reliance on training annotation (Figure 7), distinguishing our study from previous efforts in utilising CV for intraoperative guidance, which have shown limited exploration in this particular area.

### 4.1. Limitations

There are some technological limitations in our platform. An unknown object inserted in the surgical field, such as a metal clip, can interfere with landmark recognition. This issue has not been previously reported or addressed in the existing literature. The current algorithms used in surgery are supervised and primarily designed for two-dimensional analysis of a three-dimensional surgical field. However, in the presence of unknown objects that occlude the known objects, these algorithms may encounter difficulties in recognition. In this study, we have not evaluated the performance of the algorithms in cases involving occlusion by unknown objects. In addition to occlusion, there is a significant translation of the view when the microscope is moved, which impacts the tracking of the algorithm. Specifically, we plan to incorporate considerations for translation in out-of-frame surgeries and for instances where the anatomy is obscured by surgical tools. We intend to address both these issues in the next update of our algorithm. Lastly, we observed instances where frames or anatomical structures appeared blurry interfering with recognition and tracking. This blurriness can be attributed to the inherent challenges of object detection in microscopic surgery, where the lens may be out-of-focus. Additionally, anatomical structures can appear hazy due to fluid or bleeding. To mitigate these issues, we intend to enhance our model’s ability to recognise such barriers and refrain from providing anatomical predictions when these barriers are identified.

We evaluated the architecture on a single surgical video from one patient. However, to ensure the clinical applicability of the platform, it is essential to conduct further multi-centred trials involving multiple patients and various types of surgeries. However, previous research by Tokuyaso et al. has reported poor concordance among surgeons when labelling anatomical data. Therefore, to minimise bias and subjectivity (7, 26), we plan to involve multiple experts in annotating the surgical images and assess the inter-rater reliability. Additionally, it is important to note that spinal dAVF surgery is a microscopic procedure that does not currently allow our platform to provide superimposed guidance during surgery, as seen in many laparoscopic studies (7, 11, 12). To address this limitation, our research group is currently exploring an upgraded architecture that can process sparsely labelled data from multiple patients who have undergone endoscopic microvascular decompression (MVD) surgery (27).

### 4.2. Future directions

Our plans involve developing our network to overcome the technological limitations associated with identifying unknown objects, visual obstruction by tools, and out-of-focus frames (7, 11). To address the issue of unknown objects, we propose the network to adapt to and recognise previously unseen objects. For accurate tracking, our objective is to implement video-based segmentation that enables continuous tracking even when objects appear and disappear within the frame whilst retaining information about object trajectories and make more informed predictions. Furthermore, we aim to develop task-driven segmentation, as segmentation itself can be an ill-posed task, even with the availability of ground truth annotations. By implementing these advancements, we aim to address the technological limitations and enhance the performance and versatility of our network for intraoperative guidance.

In future updates, our goal is to incorporate both spatial and temporal annotation by utilizing a combination of semantic, instance, and phase recognition techniques, which were not explored in this study due to its scope limitations. By integrating these techniques effectively, we anticipate significant improvements in both the accuracy of spatial annotation and the overall understanding of surgical procedures. This expanded annotation approach holds great potential in providing valuable insights for surgery, including early error detection, surgical decision support, and performance feedback in complex neurosurgical cases. These advancements have the potential to enhance surgical care by enabling a more comprehensive analysis of surgical procedures and facilitating continuous improvement in surgical outcomes (28–30).

As demonstrated with a previous version of our architecture (27), we are currently extending our framework to be used in endoscopic surgeries that involve decompression of cranial nerves at the skull base. In the preliminary work, we conduct experiments using multiple patient videos and leverage Few-Shot Learning techniques within a sparsely labelled paradigm (27). The need for numerous expert annotations and validation poses a challenge for the generalisation of our framework across various surgical cases and specialties. Therefore, we are currently exploring different models of unsupervised learning techniques to improve the utilisation of sparsely labelled datasets and sample efficiency. With transfer learning capabilities of our CV methodology, we are actively scaling the core technology for future iterations and further development of the SMI.

Addressing these limitations provides an outlook that can have a revolutionising impact on global neurosurgery, by improving the standard of neurosurgical care and training. Our ongoing study exemplifies the foundational technology behind a SMI, a concept that aims to enhance patient outcomes and provide better training opportunities.

## Data Availability

The data that support the findings of this study are available on request from the corresponding author.
